# Prevalence of Oral Mucosal Variations in Healthy Elderly Individuals of Taxila City, Pakistan

**DOI:** 10.7759/cureus.69299

**Published:** 2024-09-12

**Authors:** Saman Malik, Faiqa Hassan, Muhammad Muddassar, Azka Haroon, Fouzia Aslam, Maria Rabbani

**Affiliations:** 1 Oral Biology, HITEC Institute of Medical Sciences (IMS) Dental College, Taxila, PAK; 2 Oral Medicine, HITEC Institute of Medical Sciences (IMS) Dental College, Taxila, PAK; 3 Oral Pathology, HITEC Institute of Medical Sciences (IMS) Dental College, Taxila, PAK; 4 Oral and Maxillofacial Surgery, Hazrat Bari Imam Sarkar (HBS) Medical and Dental College, Islamabad, PAK; 5 Community and Preventive Dentistry, HITEC Institute of Medical Sciences (IMS) Dental College, Taxila, PAK

**Keywords:** aging, mucosal variations, oral diagnosis, oral medicine, oral mucosa, pseudo lesions

## Abstract

Background and objectives

Oral mucosal variations, though significant in the aging population, remain under-researched regarding their prevalence and characteristics in local Pakistani population. This study aimed to investigate the prevalence of normal oral mucosal variations in the elderly population, providing insights to aid clinicians and researchers.

Methodology

This cross-sectional, observational, descriptive study was conducted over four months, from December 2023 to March 2024, in the Department of Oral Medicine at HITEC Institute of Medical Sciences (IMS) Dental College, Taxila, Pakistan. A total of 385 male and female patients over 60 years of age were examined. Of these, 250 patients met the inclusion criteria. All patients underwent extraoral and intraoral examinations by a consultant in dental medicine with five years of clinical experience. Diagnoses were made following the World Health Organization’s Guide to Epidemiology and Diagnosis of Oral Mucosal Diseases.

Results

Among the N = 143 female patients, approximately 33% (n = 47) exhibited age-related oral mucosal variations, while 23.4% (n = 25) of the N = 107 male patients showed such variations. The most prevalent variation was a coated tongue, found in 8.3% (n = 12) of females and 5% (n = 6) of males, followed by depapillated n = 6 (4.2%) and fissured tongues n = 3 (2.8%) respectively. Sublingual varices were the least common, occurring in 1.4% (n = 2) of females and 1.8% (n = 2) of males.

Conclusion

This study highlights the prevalence of normal age-related mucosal alterations and their gender predispositions, which can facilitate the development of future health strategies to improve oral health and promote informed care practices.

## Introduction

Literature indicates that a substantial proportion of the population, estimated to be around 60%, is affected by variations in mucosal tissue. These variations can manifest in different forms, ranging from benign alterations in texture and color to more pronounced changes that may influence oral health or contribute to systemic conditions. Understanding the prevalence and clinical implications of mucosal variation is critical for early diagnosis and management, as these variations may be symptomatic of underlying diseases or conditions that require timely intervention [1]. The oral mucosa acts as a resilient and protective barrier, shielding deeper tissues from environmental stresses that can impact oral health and, consequently, systemic health. The interplay between oral and systemic health and their bidirectional correlation can crucially impact the quality of life [2-4].

Despite its critical role, the oral mucosa is susceptible to various factors that can induce alterations in its morphology, histological features, and texture. These factors include advancing age, frictional and mechanical trauma, bacterial, viral, and fungal infections, smoking, betel nut chewing, and systemic conditions like diabetes mellitus and Sjogren’s syndrome [5-7]. The literature emphasizes the need to subcategorize these alterations into normal variations and pathological lesions, as normal variations do not require medical treatment [1].

Despite the crucial role of the oral mucosa in maintaining overall health, there is a scarcity of research delineating the prevalence and characteristics of normal variations among the elderly population [3]. Developing a consensus on the prevalence of oral mucosal variations among healthy elderly patients is essential to avoid unnecessary medications. Although a significant portion of the population (10-60%) exhibits mucosal changes with aging, this topic remains under-researched in local Pakistani population. This gap hinders dental practitioners' ability to differentiate between age-related changes and pathological conditions, potentially leading to over diagnosis and unnecessary medical interventions [1,3,8].

Recognizing the limited data on oral mucosal variations, we aimed to investigate the prevalence of normal oral mucosal variations in the local elderly population. This study provides insights that could open new avenues for clinicians and researchers in this area. We considered 60 years to be the age limit for delineating patients as elderly, as reported in the literature [9,10]. We excluded patients with degenerative diseases like hypertension and diabetes from this study because these conditions not only impact systemic health but also adversely affect the normal architecture of the oral mucosa [10,11].

Based on the limited research on the prevalence of age-related mucosal variations and the need to categorize them into normal and pathological variations, we conducted this study to determine the prevalence of normal oral mucosal variations in healthy elderly patients. This study aims to establish the relationship between mucosal variations and age, helping us differentiate normal age-related variations from pathological lesions. The findings will aid in avoiding unnecessary treatments and improve clinical decision-making.

## Materials and methods

Study design

This cross-sectional, observational descriptive study was conducted over four months, from December 2023 to March 2024, within the Department of Oral Medicine at HITEC Institute of Medical Sciences (IMS) Dental College, Taxila, Pakistan.

Sample size calculation

The sample size for this study was calculated using OpenEpi (www.openepi.com), an online application for epidemiological calculations. The calculation was based on guidelines for prevalent studies, assuming an infinite population. A confidence level of 95% and a margin of error of 5% were used, resulting in a sample size of 385.

Sampling technique

We employed a purposive sampling technique to select 385 male and female patients aged above 60 years who reported to the OPD for dental examinations and treatments in the last few months. Participation consent was obtained from all patients. Out of the 385 patients, 250 were included in the study based on their medical history.

Inclusion and exclusion criteria

Patients included in the study were those above 60 years of age, healthy with no significant medical history, and non-smokers. Excluded from the study were patients below 60 years of age, those with a history of chronic diseases such as oral lichen planus, oral potentially malignant disorders, diabetes, hypertension, heart, and kidney diseases, and smokers including persons using smokeless tobacco as well. A total of 135 patients were excluded due to the presence of chronic diseases, which could impact oral health and cause variations that may confound the results.

Ethical approval

The study was conducted following ethical approval from the Ethical Review Committee at HITEC Institute of Medical Sciences (IMS) Dental College, Taxila, Pakistan (REF: Dental/HITEC/IRB/64).

Data collection

All patients were examined by a consultant in oral/dental medicine with five years of clinical experience. The examinations were performed extra-orally and intra-orally (lips, all surfaces of tongue, gingivae, buccal mucosa, hard and soft palate) using a dental mirror under good lighting conditions. The diagnosis followed the World Health Organization’s Guide to Epidemiology and Diagnosis of Oral Mucosal Diseases [2,12]. Cotton swabs were used to clean debris and examine the lesions. The examiner used a predefined checklist to identify and document mucosal variations. This checklist was prepared after consulting oral pathologists and reviewing relevant literature. Tori were excluded from the list of examined variations.

Data recording

The variations were recorded based on the examiner's clinical judgment. No laboratory testing or biopsies were performed in this study.

Tools and instruments

The structured proforma routinely used in the dental OPD was employed to obtain patients' medical histories. The checklist given to the examiner included prevalent normal variations, excluding oral pathological mucosal lesions, as detailed in Table 1.

**Table 1:** Oral mucosal variations reported in literature

Oral variations	Prevalent normal variations reported in the literature
Fissured tongue	[1–3,11,13,14]
Crenated tongue	[11]
White and black hairy tongue	[1,13]
Coated tongue	[2,3,11,14]
Sublingual varices	[11,13–15]
Hyperpigmented mucosa	[2]
Depapillated tongue, including geographic tongue	[1,2,13]
Linea alba	[3,15,16]
Fordyce granules	[1,3,13,15]
Torus palatinus	[14]
Torus mandibularis	[14]

## Results

Data was collected from 385 male and female patients aged above 60 years. Based on demographic and health information, 250 participants (n = 143 females and n = 107 males) were found to be in good health, with no known chronic diseases, which were exclusion criteria for the study. Among these participants, approximately 33% (n = 47) of females exhibited variations in their oral mucosa related to age, while 23.4% (n = 25) of males displayed normal mucosal variations.

The detailed results of extra-oral and intra-oral examinations revealed that the most prevalent variation was a coated tongue, observed in 8.3% (n = 12)of females and 5% (n = 6) of males. This was followed by depapillated and fissured tongue, found in 4.2% (n = 6) of females and 2.8% (n = 3) of males, respectively. The lowest prevalence was seen in sublingual varices, present in 1.4% (n = 2) of females and 1.8% (n = 2) of males.

Interestingly, most mucosal variations showed a higher prevalence among females, except for sublingual varices, which were slightly more common in males. These findings provide significant evidence that oral mucosal lesions are prevalent among the elderly as normal age-related variations. This insight is crucial as it underscores the need for targeted interventions and public health initiatives aimed at improving oral health awareness and care in older adults (Table 2).

**Table 2:** Prevalence of oral mucosal variations in Taxila city

Oral Variations	Female (Prevalence)		Males (Prevalence)	
	Percentage	Number	Percentage	number
Fissured tongue	4.2%	6	2.8%	3
Crenated tongue	3.5%	5	1.8%	2
White and black hairy tongue	2.8%	4	2.9%	2
Coated tongue	8.3%	12	5%	6
Sublingual varices	1.4%	2	1.8%	2
Hyperpigmented mucosa	2.8%	4	1.8%	2
Depapillated tongue, including geographic tongue	4.2%	6	3.7%	4
Linea alba	2.8%	4	1.8%	2
Fordyce granules	2.8%	4	1.8 %	2
Total %	32.9% out of 100%	47 out of 143 females were affected	23.4 % out of 100%	25 out of 107 affected

## Discussion

The prevalence of oral mucosal variations is an important yet under-researched topic in local settings. Several etiological factors have been reported in the literature that can cause these alterations, one of which is advancing age. This study aimed to provide a deeper insight into the prevalence of these variations in the population of Taxila City resulting from advancing age to design effective preventive measures, early diagnosis, and appropriate interventions.

Age significantly affects the texture and structure of the oral epithelium, impacting its vascularity, elasticity, and ability to regenerate and repair [4,8,17,18]. Literature has reported a high prevalence of fissured tongue among elderly patients across different regions, indicating its significance as a common oral variation. For instance, one study reported the prevalence of fissured tongue to be 46%, followed by coated tongue at 20% [3], which differs from our findings. In our study, the most prevalent mucosal variation was coated tongue, contrasting with the findings of Patel and Agrawal study [3]. Our results showed 4.2% (n = 6) of females and 2.8% (n = 3) of males had fissured tongue. Given this high prevalence, dental healthcare providers must be able to recognize and understand this lesion to avoid unnecessary management for patients.

Coated tongue is another common normal mucosal variation seen among elderly patients [19]. Literature suggests its occurrence is linked to factors such as poor oral hygiene and aging-related changes in the oral epithelium. A study conducted in Costa Rica found that 54% of the elderly population had a coated tongue [14], making it the most prevalent variation among their elderly population. However, in our findings, the prevalence of coated tongue was 8.3% (n = 12) in females and 5% (n = 6) in males.

Other commonly reported normal variations of the tongue include crenated tongue, lingual varices, and depapillated tongue, which have also been observed in elderly populations [14]. Studies conducted on the Asian population show quite a difference in the prevalence of these variations across different regions. For example, the prevalence of lingual varices in India was 1.4%, whereas it was 42% in the Iranian population. Our findings were similar to those of the Indian population for males (1.8%), while the prevalence in females was slightly higher at 3.4% [3].

Linea alba, although relatively less prevalent, presents challenges in diagnosis due to its resemblance to potentially malignant lesions. Our study also exhibited lower prevalence rates of linea alba and Fordyce granules, which are still important to consider in clinical practice. We reported Fordyce spots at 1.8% (n = 2) to 2.8% (n = 4), which differs from the data reported in the literature. A study conducted in India reported its prevalence to be 6.6%, which is higher than in our study, even though both countries are in the same region of Asia [20]. Similarly, a study in Saudi Arabia reported a prevalence of linea alba around 35%, which is much higher than our findings for the population in our region [16]. These variations may have implications for oral health, highlighting the need for comprehensive oral examinations and awareness among healthcare professionals.

The possible weaknesses of this study include the use of a purposive sampling technique, which may introduce selection bias and limit generalizability. Excluding patients with known and common chronic diseases could result in missing important data on how these conditions interact with age-related mucosal variations. Conducting the study within a single institution introduces location-specific biases, and the cross-sectional design does not allow for observing changes over time. Examiner bias could be present as the examinations were conducted by a single oral and maxillofacial surgeon. Reliance on self-reported health data for excluding patients may lead to inaccuracies. The absence of laboratory testing or biopsies reduces diagnostic accuracy. The study may not have adequately accounted for confounding factors such as dietary habits, medication use, or socioeconomic status. The short study duration of four months may not capture the full spectrum of variations, and the lack of consideration for ethnic and genetic factors limits the applicability to more diverse populations. Addressing these weaknesses in future research could enhance the robustness and applicability of the findings, improving understanding and patient care strategies. We conclude that the multifaceted nature of oral health underscores the need for a comprehensive and integrative approach to address the various factors contributing to the prevalence of these lesions. Moving forward, continued research efforts are essential to refine our understanding, improve preventive measures, and enhance the overall oral health of diverse populations.

## Conclusions

In this study, females exhibited more age-related mucosal variations compared to males in the local population of Taxila city. These findings highlight that age-related mucosal variations are prevalent among the elderly population, offering a foundation for targeted interventions and public health initiatives. Common mucosal variations such as coated tongue, fissured tongue, and depapillated tongue are prevalent among patients above 60 years of age. Therefore, healthcare providers must be vigilant in recognizing and understanding the distribution of these normal oral variations to avoid misdiagnosis and unnecessary treatment, particularly in the elderly population. This approach will help in avoiding unnecessary management and ensuring the well-being of elderly patients through accurate diagnosis and appropriate care.
